# CBD’s potential impact on Parkinson’s disease: An updated overview

**DOI:** 10.1515/med-2024-1075

**Published:** 2024-10-28

**Authors:** El Ghachi Hafida, Soulimani Rachid, Gamrani Halima, Kissani Najib

**Affiliations:** Neurosciences, Pharmacology, and Environment Unit (NPEU), Faculty of Sciences Semlalia, Cadi Ayyad University, Marrakesh, Morocco; LCOMS/Neurotoxicologie Alimentaire et Bioactivité, Université de Lorraine, 57000, Metz, France; Department of Neurology, Faculty of Medicine and Pharmacy, University Hospital Mohamed VI, Medical Research Center, University Cadi Ayyad, 40000, Marrakesh, Morocco

**Keywords:** cannabidiol, Parkinson’s disease, l-DOPA-induced dyskinesia, non-motor symptoms, clinical trials

## Abstract

**Background:**

Parkinson’s disease (PD) is primarily known as a motor disorder; however, its debilitating non-motor symptoms have a significant impact on patients’ quality of life. The current standard treatment, l-DOPA, is used to relieve motor symptoms, but prolonged use is often associated with severe side effects. This creates an urgent need for effective alternatives targeting both motor and non-motor symptoms.

**Objectives:**

Over the past decade, *Cannabis sativa* and its cannabinoids have been widely studied across various health conditions. Among these compounds, cannabidiol (CBD), a non-psychoactive component, is garnering growing interest due to its multi-targeted pleiotropic properties. This work aims to provide a comprehensive overview of CBD’s efficacy in PD.

**Methods:**

This review compiles data on both motor and non-motor symptoms of PD, integrating results from preclinical animal studies and available clinical trials.

**Results:**

Preclinical research has demonstrated promising results regarding CBD’s potential benefits in PD; however, the total number of clinical trials is limited (with only seven studies to date), making it difficult to draw definitive conclusions on its efficacy.

**Conclusions:**

While preclinical findings suggest that CBD may have therapeutic potential in PD, the limited number of clinical trials highlights the need for further research. This review emphasizes the gaps that need to be addressed in future studies to fully understand CBD’s role in treating both motor and non-motor symptoms of PD.

## 1 Introduction

Parkinson’s disease (PD) is a progressive neurodegenerative disease characterized by the degeneration of dopaminergic neurons in the substantia nigra pars compacta. It affects 4.5 million people worldwide, with a prevalence of 1% of the population over 60 years old; it is the second most common neurodegenerative disease [1], accounting for more than 90% of sporadic cases, and is associated with a high lifetime cost and impairment [2]. PD is generally defined as a movement disorder characterized by several common manifestations, such as abnormal posture, bradykinesia, resting tremor, and rigidity; however, other non-motor symptoms (e.g., sleep impairment, depression, and pain) are also prominent comorbidities [3].

The pathophysiology of idiopathic PD is still not fully understood, but environmental and genetic variables are known to be involved in the disease. Head injury, age, obesity, sedentary lifestyle, rural life, and herbicide/insecticide exposure have all been linked to PD in several studies; however, smokers and coffee users have a lower risk of developing PD [4]. The pathophysiology of this disease involves multiple molecular and cellular dysfunctions, including mitochondrial dysfunction, oxidative stress, alpha-synuclein misfolding and aggregation, calcium homeostasis dysregulation, and neuroinflammation [5]. These changes may result in the death of dopaminergic neurons in the nigrostriatal pathway, which is the most severe feature of PD [6].

The main standard of medical therapy to improve the symptoms of dopamine deficiency is the dopamine precursor levodopa. However, while levodopa is highly efficacious, especially when combined with DOPA decarboxylase blocking agents such as carbidopa, which inhibit the metabolic degradation of levodopa in the peripheral nervous system before it is released into the central nervous system, chronic use may lead to reduced efficacy and difficulty in titration as the therapeutic window narrows [7]. Furthermore, antiparkinsonian treatment accounts for 22–58% of the direct costs for PD patients worldwide [1]. As a result, there is an urgent need to discover more effective and tolerable alternative options with fewer side effects to slow neurodegeneration and improve patients’ quality of life.

Recently, derivatives extracted from Cannabis (*Cannabis sativa* L.) have attracted the focus of several studies and regulations. Apart from delta-9-tetrahydrocannabinol (Δ9-THC), which is known for its psychotropic effects, there are other non-psychotropic phytocannabinoids, and cannabidiol (CBD) is the most well-known of them because it is non-intoxicating and has several advantageous pharmacological properties. Given the growing body of literature on the clinical use of CBD, PD is one of the movement disorders of interest to researchers seeking to discover its possible therapeutic potential due to its growing popularity and favorable side effect profile [7].

In Europe, Gowers described the beneficial use of *Cannabis indica* tincture for treating PD for the first time in 1888. A century later, Cannabis has gained traction as a potential PD therapy [8]. Patients with PD have been found to express high levels of cannabinoid receptors (CBRs) [9]; thus, Parkinsonian models have recently been used to study the effects of the endocannabinoid system (ECS) on basal ganglia functioning and corticostriatal processing. However, there is still a lack of clinical data on the benefits of cannabinoids in patients with PD [10].

Several lines of evidence show that dopamine (DA) depletion results in a significant rearrangement of the striatal ECS. There is a strong interaction between the endocannabinoid and dopaminergic systems through specific G protein-coupled cannabinoid receptors (CB1 and CB2) in the basal ganglia. This interaction is involved in both the control of synaptic function and the regulation of motor behavior [11]. Despite progress in the molecular understanding of how cannabinoids (CBs) interact with DA, the clinical impact of CB therapies on the motor symptoms of PD is still unclear [12].

Several recent studies have investigated the role of CBD in reducing tremors and movement impairment caused by PD. According to a theory, CBD’s possible therapeutic effects in movement disorders such as PD are thought to be due to neuroprotection and a reduction in dopaminergic neuron degeneration [13].

This work presents a comprehensive review of the literature on the use of CBD in PD. The structure of this review begins with an examination of the safety and tolerability of the CBD molecule, followed by its interaction with other cannabinoids to modulate the ECS. The main objective of this review is to present essentially all the animal and clinical studies that have used CBD to treat both motor (including l-DOPA-induced dyskinesia [LID]) and non-motor symptoms of PD and highlight gaps to be filled in future research.

## 2 CBD: a phytocannabinoid with a pharmacological profile of interest

*C. sativa* plants contain more than 500 phytochemical compounds, at least 113 of which are identified as phytocannabinoids [14]. Among this mixture of phytocannabinoids, the compound that has gained attention in recent decades, in addition to Δ9-THC, is CBD, which is known as one of the main nonpsychotropic molecules of cannabis. This phytocannabinoid constitutes up to 40% of some plant extracts [2]. CBD was originally isolated in 1940 by Adams et al. [15], after which Mechoulam and Shvo determined its structure 23 years later [16]. The CBD concentration varies greatly depending on the plant’s genotype, the growing environment, and the part used to make the extract [17]. The (−) CBD isomer is the primary naturally occurring component of *C. sativa*. To date, CBD is recognized as a pleiotropic compound that acts on multiple targets; however, its molecular pharmacology needs to be further investigated.

In terms of CBRs, CBD has a low affinity for both CB1 and CB2 receptors [2,18,19]. According to Jones and colleagues, CBD showed no CB1 receptor agonist activity but was able to inhibit cannabinoid agonists *in vitro* by acting as an inverse agonist for both receptors CB1 and CB2 [20]. It also acts as an indirect agonist at CB1R by increasing endogenous levels of anandamide (AEA) via fatty acid amide hydrolase (FAAH) blockade [21,22]. CBD is thought to have no impact on how 2-AG interacts with either CB1Rs or CB2Rs [23,24]. However, CBD acts as a negative allosteric modulator of CB1R by decreasing the efficacy and potency of 2-AG at that receptor [25]. Regarding the impact of CBD on non-cannabinoid targets, it has been demonstrated that CBD acts as an agonist of transient receptor potential vanilloid channel type 2 (TRPV-2) and an antagonist of transient receptor potential for melastatin (TRPM8) [26,27]. Additionally, according to de Petrocellis et al., CBD can indirectly agonist potential vanilloid channel type 1 (TRPV-1) receptors by increasing the levels of anandamide, an endogenous agonist of TRPV-1 [21]. In terms of dopamine neurotransmission, CBD inhibits the dopaminergic receptor [28], thereby increasing endogenous levels of dopamine [29] and acting as a negative allosteric modulator of the dopaminergic receptor D2 [30]. As a result, it is suggested that CBD may have an effect on dopaminergic neurotransmission in the basal ganglia.

Under various experimental conditions, CBD reduces the production of several molecules, such as prostaglandin E2 [31], reactive oxygen species, and inducible nitric oxide synthase [13,32,33,34] and influences the production of numerous pro-inflammatory molecules [35,36,37]. The anti-inflammatory and antioxidant properties of CBD may explain most of its neuroprotective effects. Nevertheless, the specific pathways and mechanisms through which CBD exerts its effects in PD and other neurodegenerative disorders are still under investigation.

## 3 CBD safety and tolerability

Due to its potential medical uses, CBD is currently the focus of significant investigations, especially after the Food and Drug Administration (FDA)-approved Epidiolex^®^ in 2018 for the treatment of infantile refractory epilepsy syndromes (Dravet and Lennox-Gastaut). However, CBD is not completely risk-free. In preclinical research, adverse effects of CBD on animals included hypotension, changes in organ weight, decreased spermatogenesis, neurotoxicity of the central nervous system, hepatocellular damage, developmental toxicity, and embryo-fetal death, but these toxic effects were essentially observed at high doses and above-recommended levels [38]. Clinical investigations have shown that CBD can cause liver issues, diarrhea, fatigue, vomiting, somnolence, and more importantly, CBD-induced drug-drug interactions [38]. The first comprehensive review and meta-analysis of the side effects and tolerability of CBD across all medical indications revealed that only children with epilepsy were susceptible to pneumonia, respiratory depression and aspiration, sedative effect, lower appetite, and abnormal liver function. After excluding research on pediatric epilepsy, diarrhea was the sole unfavorable effect connected to CBD therapy [39]. However, while the side effects of CBD are generally less severe compared to Δ9-THC, they should not be overlooked.

In an open-label study on PD, 100 mg/mL Epidiolex^®^ was given to patients at doses ranging from 5 to 20–25 mg/kg per day for 10–15 days. According to the study’s findings, the side effects of Epidiolex were generally minor, and none were severe [40]. However, co-administration of clobazam with Epidiolex leads to a threefold increase in the active metabolite of clobazam, consequently increasing the risk of side effects such as excessive sedation [38]. An interesting review published in 2019 discusses CBD interactions with other drugs and the resulting side effects (for more details, see [41]). CYP450 enzymes play a crucial role in the metabolism and biotransformation of most endogenous and xenobiotic compounds. CBD has been shown to interact with several isoforms of these enzymes, including 3A4, 2C9, 2C19, 1A2, 2C8, 2B6, and 2E1 [41]. For instance, this phenomenon highlights the importance of monitoring potential drug interactions, particularly in elderly patients, where medication management is already complex and this demographic is often characterized by comorbidities, polypharmacy, and physiological changes that affect pharmacokinetics and drug tolerability [42].

According to the existing evidence from clinical trials, CBD is generally well tolerated and has few major side effects compared to Δ9-THC; nevertheless, interactions with other drugs should be carefully monitored, particularly in vulnerable populations such as the elderly and patients with chronic diseases.

## 4 Modulation of the ECS with cannabinoids in PD

The ECS is an endogenous system that consists of cannabinoid receptors (CB1 and CB2), their ligands (*N*-arachidonoyl ethanolamine [AEA or anandamide] and 2-arachidonoylglycerol [2-AG]), the enzymes that synthesize (*N*-arachidonoyl phosphatidyl ethanol phospholipase D [NAPE-PLD] and diacylglycerol lipase) and metabolize them (FAAH and monoacylglycerol lipase) [43]. During the course of PD, the ECS exhibits neurochemical modifications, with CB1 receptors downregulated in the early stages and upregulated (together with CB2 receptors) with a higher endocannabinoid tone in the intermediate/late stages of the disease, according to animal and human studies [44,45,46].

The preclinical research performed over the past 20 years has demonstrated that cannabinoids (both natural and synthetic) can control neurochemical changes in the glutamatergic and GABAergic systems caused by decreased dopamine levels by activating and/or inhibiting CB1/2 receptors [34,45,46,47,48]. The anatomical and functional complexity of CB1 receptors and the distribution of endocannabinoids in several subareas of the basal ganglia are also discussed in these studies. Moreover, preclinical research suggests that CB1 receptor agonists and antagonists, as well as drugs that modulate endocannabinoid metabolism, could be useful in the treatment of this disorder [34,45,46,47,48].

**Figure 1:**
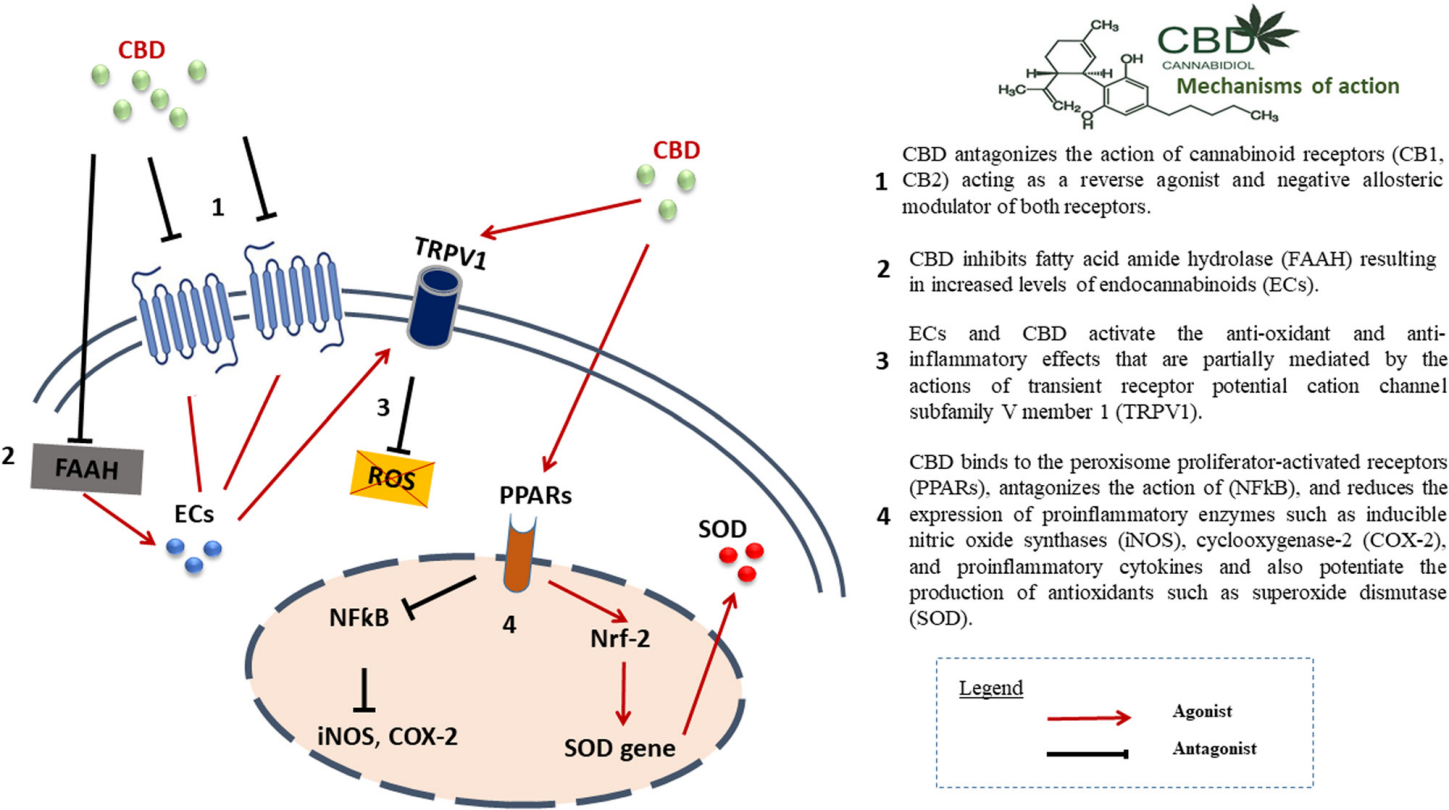
Proposed mechanisms of action of CBD in PD.

Several mechanisms (Figure 1) have been implicated in the action of cannabinoids in PD. These include antioxidant, anti-excitotoxic, and anti-inflammatory properties (mediated not only by activation of CB1R but also CB2R), inhibition of anandamide hydrolysis and their actions on other receptors, including modulation of the TRPV1 receptor channel and G protein-coupled receptor 55 (GPR55) among others [34,45,46,47].

Generally, cannabinoids act at two levels of basal ganglia function: glutamatergic/dopaminergic synaptic neurotransmission and corticostriatal plasticity, both of which are important in LID [10]. Additionally, activation of the ECS may result in neuroprotective effects via direct receptor-independent mechanisms [49], activation of anti-inflammatory cascades in glial cells via CB2Rs [34], and anti-glutamatergic anti-excitotoxic effects [47].

In fact, cannabinoids were shown to affect catecholaminergic and dopaminergic systems in early animal experiments [50,51]. Dopaminergic pathways, including the striatum, contain significant levels of CB1Rs, anandamide, and 2-AG, which regulate dopaminergic transmission via retrograde feedback mechanisms at presynaptic glutamate and GABAergic nerve terminals [52]. In the GABAergic system, it has been suggested that by blocking GABA uptake into the lateral part of the globus pallidus internus (GPi), cannabinoid agonists enhance GABAergic transmission in the indirect loop of the basal ganglia. In the glutamatergic system, the activation of CB1R at glutamate synapses inhibits the excitation of *N*-methyl-d-aspartate (NMDA) and AMPA receptors on dopamine neurons, which leads to the inhibition of excitation. Both methods may play a role in preventing dyskinesia [53].

The primary thalamo-cortical output region within the basal ganglia, particularly the medial section of the GPi, displays a high concentration of CB-1 receptors [54]. The presence of CB2Rs on human nigrostriatal dopaminergic neurons [11] shows that the ECS has direct modulatory effects on dopaminergic transmission [46]. Although nigrostriatal neurons do not express CB1R [55], they are regulated by the ECS [47,56] through CB1Rs which are expressed on GABAergic, glutamatergic, and opioidergic neurons [57]. Furthermore, interactions with various endocannabinoids have been reported for TRP channels expressed on dopaminergic neurons [58,59].

CB1R activation in the midbrain has also been shown to increase acetylcholine release, thereby ameliorating the local cholinergic deficit observed in PD [60]. Furthermore, cannabis interactions with the serotonergic system may have an impact on LID; denervation of striatal dopamine neurons causes a shift in the conversion of levodopa to dopamine from dopamine to serotonin neurons, resulting in non-physiological pulsatile dopamine release (false messengers) [61].

The concentration of CB receptors on the target structure largely determines the activating effects of a ligand with low CB receptor affinity, such as Δ9-THC. This may explain why CB ligands have such a wide range of effects, as the concentration of CB receptors varies so much in different brain areas. Moreover, the relative sensitivity of GABAergic and glutamatergic neurons to CB1-R agonists varies among species [62].

In terms of corticostriatal plasticity, experimental *in vivo* PD studies have shown that the ECS appears to influence corticostriatal synaptic plasticity. CB1R agonists reduce abnormal dopamine-mediated corticostriatal long-term potentiation and promote long-term depression in LID conditions, rendering glutamatergic synapses less responsive to future stimulation. This antidyskinetic effect is reversed by CB1R inhibition [46].

Investigations of PD animal models and human tissues from PD patients revealed an increase in ECS activity [63], with overexpression of CBRs [64,65], accumulation of cannabinoid receptor agonists [66,67], and a decrease in their breakdown [68]. In an animal model, chronic levodopa substitution reversed this ECS adaptation [69].

Overall, preclinical research has shown that cannabinoids can modulate the ECS, influence neurochemical changes in different systems, and potentially offer neuroprotective effects in PD. These findings suggest that ECS modulation could be a promising therapeutic approach. However, it is important to recognize that the translation of these preclinical findings to clinical practice remains uncertain.

## 5 Preclinical data on the use of CBD in PD

CBD has been studied extensively in preclinical investigations for a variety of neurodegenerative disorders [70]. The neuroprotective properties of CBD do not appear to depend on the direct activation of CB1 receptors [71], even though CB2 receptor involvement has been documented in specific pathological conditions [72]. The direct activity of CBD at these CBRs is still under debate. However, CBD could facilitate AEA-mediated effects by lowering FAAH activity, which is an indirect route [21].

The first preclinical study addressing CBD’s properties in PD was published by Lastres-Becker and his colleagues in 2005. The study revealed that administering 3 mg/kg CBD daily for 2 weeks reduced the depletion of dopamine and tyrosine hydroxylase in the striatum of rats injected with 6-hydroxydopamine (6-OHDA) immediately after lesion induction. The neuroprotective effect is likely mediated by cannabinoid receptor-independent antioxidant and anti-inflammatory properties [34]. However, when the treatment began one week later, there was no effect. Increased expression of Cu^2+^/Zn-superoxide dismutase mRNA, a key enzyme in endogenous oxidative stress responses, was associated with the effects of CBD [73].

In contrast, a 2016 study showed that 5 mg/kg CBD for 5 weeks did not affect dopaminergic neuron loss or motor impairments caused by 1-methyl-4-phenyl-1,2,3,6-tetrahydropyridine (MPTP) [74]. Another investigation in the same year tested different doses of CBD (15, 30, and 60 mg/kg, for 3 days) in a mouse model of LID by giving l-DOPA for 21 days after receiving 6-OHDA treatment. Although CBD alone had no impact, when it was combined with capsazepine (CPZ) (a TRPV-1 antagonist) at a dose of 30 mg/kg, it resulted in a remarkable reduction in involuntary movements induced by l-DOPA. Furthermore, this combination significantly reduced the levels of the pro-inflammatory markers cyclooxygenase-2 (COX-2) and nuclear factor-kappa B (NF-κB). CB1 and peroxisome proliferator-activated type gamma (PPAR) receptor antagonists prevented all of these effects. Thus, the anti-inflammatory effects mediated by CB1 and PPAR receptors are thought to be responsible for the neuroprotective effects of CBD (when combined with a TRPV-1 antagonist) [75]. CB2R activation, but not CB1R activation, may have neuroprotective effects on PD, which could be due to an anti-inflammatory effect [64,73]. CB2R agonists protected mice from MPTP-induced nigrostriatal degeneration by decreasing microglial activation and infiltration, according to rodent PD model research [76]. More recently, Wang and colleagues have demonstrated that the administration of 100 mg/kg CBD by oral gavage for 14 days alleviates PD symptoms in the MPTP mouse model. The study reported an improvement of cognitive behaviors, an increase in DA, 5-HT, and IL-10 levels, and a decrease in several apoptosis and neuroinflammation markers [77].

Drug-induced catalepsy tests, which are often used to assess motor deficits due to changes in striatal function, were employed in two investigations [78,79]. The first study aimed to test the effects of acute CBD pretreatment (5, 15, 30, or 60 mg/kg) on catalepsy caused by haloperidol (a D2 receptor antagonist), l-nitro-*N*-arginine (a selective nitric oxide synthase [NOS] inhibitor), and WIN55,212 (a CB1 receptor agonist) in male Swiss mice. CBD reduced the increase in catalepsy time caused by all three medications in a dose-dependent manner [78]. In the second study, rats were given reserpine (an irreversible inhibitor of vesicular monoamine transporters 1 and 2 or VMAT1/2) to cause motor impairments and cognitive abnormalities to imitate both tardive dyskinesia and PD. In the discriminative avoidance task, CBD doses of 0.5 or 5 mg/kg considerably reduced reserpine-induced catalepsy and chewing movements (but not locomotor activity), and the lowest dose also significantly improved reserpine-induced memory/learning deficits [79].

CBD has been shown to protect neurons in both cell tissue and animal models of PD *in vitro*. CBD enhanced the survival, differentiation, and expression of axons (GAP-43) and the synaptic proteins synaptophysin and synapsin I in a cellular model of PD in PC12 and SH-SY5Y cells treated with 1-methyl-4-phenylpyridinium (MPP+) [80]. After incubation with lipopolysaccharide (LPS), CBD enhances cell viability and decreases microglial activation [81,82]. Since chronic inflammation is a prominent hallmark of PD and dopaminergic neurons are particularly sensitive to glial activation [83], the anti-inflammatory properties of CBD may contribute to its neuroprotective potential in PD [2]. In a cell culture model of PD, differentiated SH-SY5Y neuroblastoma cells were exposed to three toxins that model PD-associated biochemical abnormalities: reduced mitochondrial activity by MPP+, free radical production by paraquat, and inhibition of the ubiquitin‒proteasome system by lactacystin. MPP+ and lactacystin administration resulted in substantial upregulation of CB1 receptors. In this model, no protective effects of CBD (0.01–1.0 M) were identified [49].

In these animal and cellular models of motor, biochemical, and cognitive symptoms, the preclinical data discussed above suggest that CBD generally alleviates PD-related symptoms. However, the majority of studies have employed a single model of motor symptoms, with only a few examining additional elements of PD. Furthermore, the overall volume of investigations is still limited, and their methodology varies in terms of CBD dosage.

## 6 Clinical trials on the use of CBD in PD

To date, 17 clinical studies using Cannabis or its derivatives to treat PD have been reported in the literature (Appendix). Seven studies utilized CBD as the main drug of interest, six studies used pure CBD [60,84,85,86,87,88], and one study used a mixture of 1.25 mg CBD and 2.5 mg Δ9-THC (Cannador^®^) [89].

### 6.1 Motor symptoms

Among all the clinical trials that have been conducted thus far investigating the usefulness of CBD in PD patients (four studies) concluded that no improvement was noted regarding the effect of CBD on the severity of motor symptoms, as evaluated by the Unified Parkinson Disease Rating scale (UPDRS) [84,86,88,89]. The details of each study are summarized in Table 1.

**Table 1:** Studies with CBD addressing motor symptoms in PD

Symptom	Design	Sample size	Duration	Primary outcome	Application form	Dosage	Outcome	References
Motor symptoms	Double-blind, randomized, placebo-controlled crossover study	17	2 × 4 weeks, 2 weeks wash-out phase	UPDRS	Orally	2.5 mg THC + 1.25 mg CBD (Cannador) or placebo	No positive effect compared with placebo	Carroll et al. [89]
	Open-label pilot	6	4 weeks	UPDRS total	Orally	150–400 mg/day CBD or Placebo	CBD did not worsen the motor function and decreased the total scores of UPDRS	Zuardi et al. [165]
	Randomized, double-blind, placebo-controlled study	21	6 weeks	UPDRS total	Orally	1. Group: CBD 75 mg/day	UPDRS unchanged	Chagas et al. [60]
						2. Group: CBD 300 mg/day		
						3. Placebo group		
	Phase II/III, randomized double-blind, placebo-controlled clinical trial	33	14 weeks	UPDRS	Orally	75 or 300 mg/day CBD or placebo	No significant difference between groups	de Almeida et al. [86]

### 6.2 Pain

In medicine, pain is a complex and multifaceted notion, making it particularly challenging to comprehend and treat therapeutically. Pain is assumed to be a combination of a subjective psychophysical experience, objective sensory neurophysiology, and emotional response to stressful stimuli [90]. In PD, chronic pain is one of the most common non-motor symptoms in PD patients, affecting 60–80% of individuals [91]. According to a study of nearly 2,000 PD patients, chronic pain cannot be explained uniquely by peripheral variables because central causes appear to play a significantly greater role than previously thought [92].

For the management of chronic pain, a range of analgesic medicines have been created and widely used. Standard medical therapy for persistent neuropathic pain includes antiepileptic medications, selective serotonin reuptake inhibitors (SSRIs), nonsteroidal anti-inflammatory drugs (NSAIDs), and in refractory situations, opioids, all of which have different efficacies and distinct long-term side effects [7].

Natural cannabinoids have emerged as strong candidates for pain therapy in the face of rising opiate misuse rates [43]. Although cannabis has been used to alleviate pain since 5000 BC, little is known about its mechanisms of action [93]. There are still some doubts about whether cannabis can help with specific forms of pain [94]. Currently, nabiximols mouth spray (Sativex^®^), an oromucosal spray containing a 1:1 ratio of CBD and Δ9-THC, is the only approved cannabis-based treatment for MS-related spasticity and neuropathic pain in some countries[95]. CBD has been shown to help with pain alleviation in several preclinical studies [96,97,98]; however, recent clinical trials evaluating the efficacy of CBD alone in other forms of pain are reporting heterogeneous results [99,100,101,102], with the lack of those focusing on Parkinson-related forms of pain.

According to our literature search, one study has assessed pain in PD patients (Table 2) using Cannador^®^ with 2.5 mg of Δ9-THC and 1.25 mg of CBD [89]. This double-blind randomized, placebo-controlled crossover study in 2004 reported no positive effect on pain assessed by the McGill Pain Scale in 17 patients. Therefore, evaluating CBD’s efficacy treatment exclusively in this population could be of interest.

**Table 2:** Studies with CBD addressing pain symptoms in PD

Symptom	Design	Sample size	Duration	Primary outcome	Application form	Dosage	Outcome	References
Pain	Double-blind, randomized, placebo-controlled crossover study	17	2 × 4 weeks, 2 weeks wash-out phase	McGill Pain Score	Orally	2.5 mg THC + 1.25 mg CBD (Cannador) or placebo	No positive effect on pain	Carroll et al. [89]

### 6.3 Depression

Depression is one of the most commonly reported neuropsychiatric disorders in PD and it is widely acknowledged that 40–50% of patients with PD experience clinically significant depressive disorders [103]. Several studies suggest that depression can occur at any stage of the disease; importantly, affective disorders often precede the onset of motor symptoms, on average 4–6 years before the diagnosis of PD [104]. These disorders may have a long-term or recurrent course and therefore contribute to the alteration of the quality of life of PD patients [105].

CBD is hypothesized to affect depression due to its capacity to target and modulate serotonin and norepinephrine brain neurotransmission as well as its active binding to 5HT-1A receptors [106]. Furthermore, CBD promotes synaptic plasticity and neurogenesis, both of which are important in the development and treatment of depression [21,52,106]. The conclusions of certain animal models have been positive and encouraging [107,108]. However, only a few clinical studies involving individual patients with a history of depression who successfully tried CBD products have been reported [107,109]. These studies should be approached with caution. CBD use has been linked to a number of negative side effects, including increased depression and even suicidal thoughts [41]. Depression and suicidal ideation are listed as possible side effects of the FDA-approved form of CBD Epidiolex’s box insert [110].

According to our literature search, there was one clinical trial (Table 3) in which CBD was administered to 10 depressed PD patients in a study including 33 patients with rapid eye movement disorder for 14 weeks, but no positive effect on depression was noted [86]. As a result, more large-scale trials are required to evaluate both the potential antidepressant benefits of CBD as well as its long-term efficacy and safety.

**Table 3:** Studies with CBD addressing depression symptoms in PD

Symptom	Design	Sample size	Duration	Primary outcome	Application form	Dosage	Outcome	References
Depression	Double-blind, randomized, placebo-controlled study	10/33	14 weeks	Zung Self-Rating Depression Scale (SDS)	Orally	CBD 75 mg/day or 300 mg/day or placebo	No significant effect between groups	de Almeida et al. [86]

### 6.4 Sleep disturbances

Sleep disorders affect 50–70 million people in the United States, resulting in millions of physician visits each year. There are numerous reviews and studies on the effects of cannabis and cannabinoids on sleep; a meta-analysis revealed significant improvement in sleep quality in 8 trials and in sleep disturbance in three trials, but these improvements were regarded as minor [95,111,112,113].

Human investigations on the effects of CBD on sleep disorders are limited and modest in number [60,88,114,115]. Sleep problems, such as poor sleep quality, insomnia, restless legs syndrome, and rapid eye movement sleep behavior disorder, are more common in PD patients [116]. Sleep problems in PD patients, similar to other non-motor symptoms, appear to be related to dopaminergic neurotoxicity and subsequent neurochemical abnormalities in areas of the brain involved in the regulation of the sleep-wake cycle mediated by cholinergic, GABAergic, and serotonergic neurons [60]. The use of benzodiazepines and, less commonly, melatonin and cholinesterase inhibitors to treat sleep disorders in this population is limited because of side effects. Benzodiazepines are associated with somnolence and impaired motor coordination, which may worsen sleep and motor symptoms in PD patients, as well as tolerance, abuse/dependence, and withdrawal symptoms. The administration of melatonin can cause somnolence and psychosis, which can increase sleep and psychotic symptoms, whereas cholinesterase inhibitors might cause gastrointestinal problems, anorexia, and bradycardia [60,117]. Studies about CBD’s influence on sleep are still in their early stages and it is assumed to influence sleep because endocannabinoids have been proven to play a role in the circadian rhythm [88,118,119].

According to our literature search, there are four studies investigating CBD on sleep quality in PD patients (Table 4). The first study was conducted in 2004 by Carroll et al. in a double-blind randomized placebo-controlled crossover study in 17 PD patients using Cannador^®^ (2.5 mg Δ9-THC + 1.25 mg CBD). The study concluded that no positive effect on sleep was detected by the visual analog sleep scale [89]. However, in 2014, Chagas et al. confirmed that 4 PD patients with REM sleep disorder experienced rapid and substantial reductions in agitation, limb movements, and nightmares, with pure CBD [60]. The third study was the first randomized, double-blind, placebo-controlled clinical trial by de Almeida et al. evaluating CBD’s effect on sleep disorders in PD patients. They concluded that prolonged use of CBD did not produce any significant difference from the placebo for primary outcomes [86]. More recently, the same results were observed in an exploratory study of phase II/III clinical trial assessing the effect of 75–300 mg of CBD in PD patients with restless leg syndrome and REM sleep disorder [87]. Therefore, there is a need for large-scale trials to assess the efficacy of CBD in the treatment of sleep disorders and its long-term safety, with the recommendation of investigating the addictive potential of CBD in long-term studies.

**Table 4:** Studies with CBD addressing sleep disturbance symptoms in PD

Symptom	Design	Sample size	Duration	Primary outcome	Application form	Dosage	Outcome	References
Sleep	Double-blind, randomized, placebo-controlled crossover study	17	2 × 4 weeks, 2 weeks wash-out phase	Visual analog sleep scale	Orally	2.5 mg THC + 1.25 mg CBD (Cannador) or placebo	No positive effect on sleep	Carroll et al. [89]
	Observational case series	4	6 weeks	PD patients with REM sleep disorder	Orally	75 or 300 mg CBD	Reduction of agitation, limb movements	Chagas et al. [84]
				Clinical assessments not mentioned			And nightmares in all patients (symptoms returned after interrupted treatment)	
	Phase II/III, randomized double-blind, placebo-controlled clinical trial	33	14 weeks	Frequency of nights with RBD^a^, CGI^b^	Orally	75 or 300 mg CBD or placebo	CBD showed no difference to placebo for primary outcomes	de Almeida et al. [86]
							Significant improvement in average sleep satisfaction with 300 mg was noted	
	Phase II/III, a parallel, double-blind, placebo-controlled clinical trial	6/18	14 weeks	Restless Legs Syndrome Rating Scale	Orally	75 or 300 mg CBD or placebo	No reduction in the severity of RLS^c^ in PD patients with RBD^a^	de Almeida et al. [87]

^a^Rapid eye movement sleep behavior disorder.

^b^Clinical Global Impression scale.

^c^Restless leg syndrome.

### 6.5 Psychosis

Psychosis is very common in people with PD, affecting almost one-third of individuals, especially in the later stages of the disease [116]. Antiparkinsonian drugs, dopaminergic neurotoxicity, and Lewy body pathology all appear to play a role in the pathogenesis of psychosis in PD. Treatment of psychosis in PD patients is complicated and remains a therapeutic challenge, as it usually involves lowering or eliminating antiparkinsonian drugs (which might worsen symptoms) and/or adding conventional antipsychotics to the regimen (which can worsen motor symptoms). Atypical antipsychotics, such as clozapine, are not linked to worsening motor symptoms, but they can have serious hematological, cardiovascular, and neurological adverse effects [88,120,121].

Several reviews [120,122,123,124,125] have shown that animal and human studies consistently suggest that CBD has antipsychotic effects and is well tolerated in general. However, there are few published clinical trials including CBD given to psychotic patients, and most of them had small sample sizes and short durations, focused on symptomatic or first-episode patients, and did not particularly address negative/cognitive symptoms [117].

The mechanisms underlying the antipsychotic activity of CBD are not yet understood, but CBD has shown a pattern of activation of Fos-immunoreactive neurons similar to that of the atypical antipsychotic clozapine but different from that of the typical antipsychotic haloperidol, with activation of limbic but not motor areas [126]. The antipsychotic effects of CBD appear to be mediated through the ECS via the inhibition of FAAH and subsequent increase in anandamide levels, as well as the activation of vanilloid TRPV1 and serotonin 5-HT1A receptors [22,120].

According to our literature search, there was one study assessing the effect of CBD on psychosis in PD patients (Table 5). This open-label pilot study demonstrated that 6 PD patients had a significant decrease in their psychotic symptoms[88]. The size of these studies is often small, and the follow-up is either brief or limited to a single event. The number of accessible research is quite restricted, and the majority of them are published as individual case reports. As a result, large-scale clinical research is required to determine CBD’s long-term efficacy and safety.

**Table 5:** Studies with CBD addressing psychosis symptoms in PD

Symptom	Design	Sample size	Duration	Primary outcome	Application form	Dosage	Outcome	References
Psychosis	Open-label pilot study	6	4 weeks	Brief Psychiatric	Orally	150 mg CBD starting dose, weekly dosage increase by 150 mg depending on clinical response	Significant decrease of psychotic symptoms	Zuardi et al. [88]
				Rating Scale, Parkinson				
				Psychosis				
				Questionnaire				

### 6.6 Anxiety

Anxiety disorders encompass a wide range of symptoms and manifestations, all of which can negatively affect one’s quality of life and capacity to perform daily tasks. These disorders are the most common mental condition with a lifetime prevalence of 29% in the general population, and the most common psychological symptoms in people with PD affecting nearly 67% of PD patients [127,128]. Although psychotherapy, SSRIs/serotonin-norepinephrine reuptake inhibitors, benzodiazepines, monoamine oxidase inhibitors, and/or tricyclic antidepressants are usually the mainstays of treatment for this group of disorders, all of these pharmacologic solutions have highly variable rates of efficacy as well as their own risk of side effects. In PD, first-line anxiolytic drugs (typically SSRIs) have low efficacy in PD patients and might worsen motor symptoms [85,116,117]. As a result, there is a growing demand for alternative pharmacologic therapies that can provide more consistent relief with fewer side effects. Consequently, CBD has lately been identified as a promising treatment option for anxiety disorders by researchers [7,127].

CBD has been shown to be a potent activator of the 5-HT1A and 5-HT2A receptors, both of which are targets of existing anxiolytic drugs such as buspirone for the treatment of generalized anxiety disorder [129]. Overall, CBD’s promise as an effective treatment for anxiety-related disorders is supported by some of the best data currently available on CBD-related research. However, as most studies have had small sample sizes and have largely investigated only acute treatment with CBD for anxiety, further research is needed to confirm this evidence and determine whether CBD is a viable option for chronic therapy in anxiety-related conditions [7].

According to our literature search, there was one study investigating the effect of CBD in PD patients with anxiety symptoms (Table 6). A randomized, double-blinded, placebo-controlled, crossover clinical trial with a total of 24 PD patients was conducted by de Faria in 2020. The findings revealed that a dose of 300 mg of pure CBD attenuated the anxiety experimentally induced by the Simulated Public Speaking Test (SPST) [130].

**Table 6:** Studies with CBD addressing anxiety symptoms in PD

Symptom	Design	Sample size	Duration	Primary outcome	Application form	Dosage	Outcome	References
Anxiety	Randomized, double-blind, placebo-controlled crossover	24	Two experimental sessions within a 15-day interval	1. 2 SPST^a^ sessions	Orally	1. CBD group: 300 mg of pure CBD dissolved in corn oil	CBD attenuated the anxiety experimentally induced by the SPST^a^	De Faria et al. [85]
				2. UPDRS^b^		2. Placebo group: corn oil capsules		
				3. VAMS^c^				
				4.SSPS^d^				
				5. Blood pressure and heart rate				

^a^Simulated Public Speaking Test.

^b^Unified Parkinson Disease Rating Scale.

^c^Visual Analog Mood Scales.

^d^Self- Statements during Public Speaking Scale.

### 6.7 CBD and quality of life in PD patients

The non-motor PD symptoms mentioned above (psychosis, anxiety, depression, sleep disturbance, and pain) are associated with PD patients’ quality of life (QOL). These symptoms are significant predictors of declining QOL [84,117]. However, few studies have examined the impact of pharmacological therapies on PD patients’ QOL, and the results are inconclusive.

The positive impact of CBD on the quality of life of PD patients may be due to its therapeutic effects on non-motor symptoms. CBD differs from traditional drugs, which usually target specific sites of action to treat specific conditions by acting on a broader range of conditions simultaneously and through different yet unidentified mechanisms of action [71,129]. The multi-target properties of CBD may make it more useful than other drugs for people with PD, as the pathophysiology of PD (and other disorders) is generally multifactorial.

According to our literature search, three studies investigated the effect of CBD on quality of life in PD patients (Table 7). The first study in 2004 with Cannador^®^ (2.5 mg Δ9-THC + 1.25 mg CBD) revealed no positive effect on quality of life. However, in 2014, Chagas et al. conducted an explorative, randomized double-blind, placebo-controlled study for 6 weeks in 21 PD patients and concluded that CBD (300 mg) significantly improved QOL [84,89]. Conversely, a recent study concluded that even doses of 75 or 300 mg CBD had no significant effect on quality of life as a secondary outcome [86].

**Table 7:** Studies with CBD addressing quality of life in PD

Symptom	Design	Sample size	Duration	Primary outcome	Application form	Dosage	Outcome	References
QOL	Double-blind, randomized, placebo-controlled crossover study	17	2 × 4 weeks, 2 weeks wash-out phase	PDQ-39^a^	Orally	2.5 mg THC + 1.25 mg CBD (Cannador) or placebo	No positive effect on quality of life	Carroll et al. [89]
	Explorative, randomized, double-blind, placebo-controlled	21	6 weeks	PDQ-39^a^	Orally	75 or 300 mg CBD or placebo	Significant improvement of quality of life (300 mg CBD group)	Chagas et al. [84]
	Randomized double-blind, placebo-controlled clinical trial	33	14 weeks	PDQ-39^a^	Orally	75 or 300 mg CBD or placebo	No significant effect between groups	de Almeida et al. [86]

^a^Parkinson’s Disease Questionnaire-39.

## 7 Effects of CBD on LID

### 7.1 LID: a brief introduction

l-3,4-Dihydroxyphenylalanine (l-DOPA), the amino acid precursor of dopamine, is still the mainstay treatment for PD motor symptoms. However, the development of debilitating motor problems such as LIDs limits its long-term efficacy. The LID is a collection of debilitating involuntary movements that includes chorea, ballism, dystonia, and, to a lesser extent, myoclonus (a rapid and involuntary muscular contraction that occurs primarily in the extremities) [131].

Treatment with l-DOPA in the early stages of PD causes “positive” plasticity, which leads to long-term symptom relief [132,133]. However, as the disease progresses, l-DOPA-induced clinical relief disappears. l-DOPA impairs striatum-dependent learning capabilities [134,135] and has a deleterious impact on cortical plasticity [136]. LIDs are linked to corticostriatal overactivity as well as molecular alterations in the basal ganglia. Striatal neurons combine cortical and thalamic inputs to adjust the basal ganglia’s output, making them important in movement selection and adaptive motor control [137]. The pathogenesis of LID has also been linked to increased glutamatergic neurotransmission [138]. The currently available pharmacological treatment for LID is amantadine, a non-competitive antagonist of NMDA-type glutamate receptors [139].

Preclinical studies have demonstrated that drugs targeting various neurotransmitters, such as noradrenaline, acetylcholine, serotonin, adenosine, and nitric oxide, can reduce LID [140,141,142].

### 7.2 Modulation of the ECS to treat LID

There is a growing body of evidence indicating that ECS is involved in dyskinesia. Several studies have investigated whether ECS modulation could be used to help people with l-DOPA-induced aberrant involuntary movements (AIMs) [2]. Some dysregulated metabolites, including AEA and 2-AG, were found in the striatum of dyskinetic rats according to a recent study [143]. Also, in hemiparkinsonian rats, the intrastriatal injection of both endocannabinoids before l-DOPA treatment delayed the start of LID, according to the same study [143].

Modifications of the CB1 and CB2 receptors suggest a potential therapeutic target for the active period of LID [144]. In support of this theory, the CB1 agonist nabilone has been shown to reduce LID in MPTP-lesioned non-human primates treated with l-DOPA [145]. In hemiparkinsonian rats, small dosages of HU-210 (a synthetic CB1 agonist) significantly attenuated l-DOPA and apomorphine-induced contralateral rotations [146]. In neurotoxic 6-OHDA-lesioned rats that were repeatedly administered levodopa, the cannabinoid agonist WIN-55,212-2 provided antidyskinetic effects, and its activity was reversed by the CB1 antagonist rimonabant SR141716A [147]. In MPTP-lesioned marmosets, coadministration of l-DOPA (8 mg/kg) and the CB1 receptor antagonist rimonabant (1 and 3 mg/kg) reduced the severity of LID without reducing the antiparkinsonian effect of l-DOPA [67]. This treatment also reduced LID in rats, with partial preservation of dopaminergic cells [148]. In MPTP-treated rhesus monkeys, CB1 blockade increased the l-DOPA response to motor impairment [149]. When TRPV1 receptors were blocked, FAAH inhibitors were found to have anti-dyskinetic effects, suggesting that CB1 and TRPV1 receptors may play opposing roles in LID [48].

In LID therapy, CB1 receptor agonists as adjuvants are theoretically more favorable than DA substitutes. CB1 receptor stimulation reduces striatal glutamate release, inhibits D1-receptor-mediated effects, and increases GABA levels in the lateral or external segment of the globus pallidus, all of which help to reduce LID [150]. However, there are conflicting results regarding the effects of CB1 activation on LID pathogenesis. When hemiparkinsonian mice lacking CB1 receptors were given l-DOPA, they developed mild dyskinesia rather than severe dyskinesia, according to Pérez-Rial and colleagues [151].

In summary, LID alleviation following CB1 pharmacological manipulation has been described in the majority of publications [2]. Blocking CB1 receptors is presumably only beneficial in specific circumstances, such as when CB1 receptor antagonists are administered at low doses in moderate parkinsonism, when patients are intolerant to dopamine therapy, or when they are in the late stages of PD [152,153]. Although these results were obtained from treatments with varying specificities and different animal models, they demonstrated that CB1 antagonists have no conclusive effect on treating PD symptoms [154].

Many investigations have confirmed the importance of CB1 in LID using nonselective agonists of CB receptors as a pharmacological tool but have ignored the potential function of CB2 in the pathophysiology of this condition [2].

### 7.3 CBD on LID: preclinical studies

To the best of our knowledge, the effect of CBD on LID has been studied in two preclinical studies [75,155]. The first investigation showed that CBD (10, 30, and 60 mg/kg) had no anti-dyskinetic effect. However, both of the two studies have confirmed the anti-dyskinetic effect of CBD when combined with TRPV-1 antagonist CPZ in hemiparkinsonian mice chronically treated with l-DOPA.

When CB1 and TRPV-1 receptors are coexpressed in the same cell or in close neuron-neuron/neuron-glia contacts, mutual modulation is thought to occur (between CB1 and TRPV-1) [156,157]. Activating CB1 and TRPV-1 receptors have shown conflicting effects on excitatory and inhibitory neurotransmission in hippocampal neurons [158]. The simultaneous activation of CB1 and TRPV-1 has different effects on intracellular calcium concentrations [159]. According to one theory to explain these findings, TRPV-1 activation by either AEA (indirectly boosted by CBD) or CBD enhances LID or impairs the positive effects mediated by other pathways, such as the stimulation of CB1 and PPAR receptors. The favorable results observed with the administration of a powerful FAAH inhibitor and TRPV-1 antagonist (arachidonoyl serotonin) support this idea since the particular increase in AEA levels (in combination with TRPV-1 receptor antagonism) reduced LID manifestations [75]. AM251 reduced the antidyskinetic effects of CPZ + CBD on limb and orofacial LID, but GW9662, a PPAR antagonist, uniquely inhibited the antidyskinetic effect on axial AIMs, corroborating these results. As a result, direct (or indirect) activation of CB1 and PPAR (together with TRPV-1 inhibition) could be a potential method for alleviating LID [2].

### 7.4 Clinical studies on the effects of CBD on LID

To the best of our knowledge, no clinical investigation has been conducted with the explicit goal of evaluating CBD’s effects on LID reduction in PD patients.

Only three randomized controlled clinical trials investigated the efficacy of other cannabinoids rather than CBD (nabilone, THC/CBD, rimonabant) in alleviating LID symptoms. Two of them concluded that no changes or improvements have been noticed neither with rimonabant nor with THC/CBD mixture treatments [89,160]. However, a study with nabilone (smoked) showed a reduction in LID severity and its duration in seven PD patients [161]. In a survey of 339 PD patients in the Czech Republic, 25% reported taking cannabis with meals in an oral formulation of fresh or dried leaves, 46% reported mild or significant relief from PD symptoms in general, 31% reported improvement of rest tremor, 45% reported relief from bradykinesia, and 14% reported improvement of LID [162]. However, after being treated with oral doses of CBD (100–600 mg/day over 6 weeks) along with standard medication, a preliminary open pilot study showed an aggravation of parkinsonian symptoms in 2 PD patients with dystonic movement disorders [163].

These few studies that have examined the effects of cannabis/cannabinoids on motor dysfunctions associated with PD (such as LID) have produced contradictory results (for more details, see Appendix).

## 8 Conclusion

The above-cited preclinical studies collectively suggest that CBD is a promising candidate for the treatment of various health conditions which is due to the fact that it lacks psychoactive effects and is a potent anti-inflammatory and antioxidant compound. In the context of clinical trials, it is challenging to derive definitive conclusions about the efficacy of CBD as a treatment for PD, given that only seven studies have been conducted thus far. Nevertheless, the current body of research is constrained by several limitations. A notable limitation is the insufficient number of large-scale, high-quality randomized controlled trials examining the efficacy and safety of CBD in the treatment of PD. The majority of existing studies are relatively small and have various methodological shortcomings, with considerable heterogeneity in terms of study design, CBD dosages, routes of administration, and outcome measures. Furthermore, the majority of patients diagnosed with PD are of an advanced age. Consequently, age-related factors, including comorbidities, polypharmacy, and alterations in pharmacokinetics and pharmacodynamics, can potentially impact the efficacy of CBD treatment. Moreover, the impact of sex differences on the effectiveness and safety of CBD has not been sufficiently explored, and male and female patients may exhibit variability in their response to treatment. Additionally, many studies do not stratify results based on disease stage, which limits the ability to ascertain the efficacy of CBD at different stages of the disease.

To address the aforementioned limitations, it is proposed that several avenues for future investigation be explored. The necessity for large-scale, double-blind, placebo-controlled trials is paramount for the evaluation of the efficacy of CBD in treating both motor and non-motor symptoms of PD. Longitudinal studies are essential to investigate the long-term effects of CBD on human health, including an assessment of its safety, efficacy, and potential disease-modifying properties. The establishment of standardized protocols for CBD dosage, administration routes, and outcome measures will facilitate greater comparability between studies and help to reduce heterogeneity. To gain a deeper understanding of the mechanisms through which CBD interacts with the ECS and other neurotransmitter systems in the context of neurodegenerative disorders, further mechanistic studies are required. An investigation into the potential advantages of combining CBD with other pharmacological interventions, such as l-DOPA, may elucidate synergistic effects that could enhance therapeutic efficacy. Furthermore, future studies should concentrate on age-specific analyses to address age-related factors and guarantee applicability to the typical demographic affected by PD. It would be beneficial to include sex-based analyses to identify any differential effects of CBD between male and female patients. Ultimately, stratifying patients based on the stages of PD will facilitate the determination of the efficacy and safety of CBD at various stages of disease progression, thereby providing more precise guidance for its use in clinical practice.

Notably, due to CBD’s well-documented safety profile in patients, it may represent a promising add-on therapy with the potential to alleviate some symptoms. Accordingly, we believe that more extensive, well-designed preclinical and clinical research addressing motor and non-motor symptoms of this disease is required to establish the true effects of CBD.

## Abbreviations

ABC: Activities of balance confidence
ADL scale: Activities of daily living
BAI: Beck Anxiety Inventory
BDNF: Brain-derived neurotrophic factor
BPRS: Brief Psychiatric Rating Scale
CGI-I: Clinical Global Impression Improvement
ESS: Epworth Sleepiness Scale
FAB: Frontal assessment battery
FSS: Fatigue Severity Scale
GNDS: Guy’s Neurological Status Scale
IPAQ: International Physical Activities Questionnaire
H1-MRS: Proton magnetic resonance scans
H&Y scale: Hoehn & Yahr
LID: (l-DOPA)-induced dyskinesias
MMSE: Mini-mental status examination
NHP: Nottingham Health Profile
NR: Nonreported
NSAID: Nonsteroidal anti-inflammatory drug
PAS: Parkinson Anxiety Scale
PDQ-39: Parkinson’s Disease Questionnaire-39
PPQ: Parkinson Psychosis Questionnaire
PRI: Pain Rating Index
PSQI: Pittsburgh Sleep Quality Index
PSS: PD Sleep Scale
RBD: Rapid eye movement sleep behavior disorder
RBDSQ: REM Sleep Behavior Disorder Screening Questionnaire
RLS: Restless leg syndrome
SDS: Zung Self-Rating Depression Scale
SPST: Simulated Public Speaking Test
SSPS: Self- Statements during Public Speaking Scale
THC: Tetrahydrocannabinol
UPDRS: Unified Parkinson Disease Rating Scale
UKU: Udvalg for kliniske undersøgelser
VAS: Visual Analog Scale
VAMS: Visual Analog Mood Scales

## Appendix Total number of conducted studies with cannabis/cannabinoids in PD, randomized controlled, and uncontrolled trials

**Table A1:** Randomized controlled trials

References	Study design	Sample size	Study duration	Type of the drug and route of administration	Measures	Main findings	Adverse events
		Age					
		Disease duration					
Sieradzan et al. [161]	Randomized, double-blind, placebo-controlled	*N* = 7	Single exposure of nabilone or placebo; half of dose administered 12 h and 1 h, respectively, before acute levodopa challenge test; crossover after 2 weeks	1. Group: smoke Nabilone (0.03 mg/kg) + l-DOPA (0.03 mg/kg) 2. placebo: l-DOPA (0.03 mg/kg)	1. Rush Dyskinesia Disability Scale	Reduction in LID severity and duration	Mild sedation, “floating sensation,” dizziness, hyperacusis, partial disorientation, and formed visual hallucinations
		Age: NR			2. H&Y	No changes were observed in the severity of PD symptom	
	Crossover study	Dd: NR			3. Modified Webster Scale		
**Carroll et al.** [**89**]	**Randomized, double-blind, placebo-controlled** **Crossover study**	***N*** **= 17** **Age: 67 years** **Dd: 14 years**	**4 weeks**	**1. Group: Cannador (1.25 mg CBD and 2.5 mg THC)** **2. Placebo: synthetic oil capsules**	**1.UPDRS**	**No improvement in LID severity duration, motor** **Symptoms, quality of life, pain or sleep**	**Mild physical and psychological symptoms**
					**2. Rush scale**		
					**3. Bain scale**		
					**4. Tablet arm drawing task**		

					**5. ADL scale**		
					**6. PDQ-39**		
					**7. On-off diaries**		
					**8. Category rating scales**		
Mesnage et al. [160]	Randomized, double-blind, placebo-controlled study	*N* = 24Age: NRDd: NR	16 days	1. Rimonabant (20 mg/day, *n* = 4) for 16 d	1. UPDRS	No change in motor impairment or LID in neither	None
				2. SR 48692, (180 mg/day, *n* = 4) for 9d			
				3. SR 142801 (200 mg/day, *n* = 4) for 19 d	2. Standardized videotape	On- nor Off-state	
				4. placebo (n = 12)			
**Chagas et al.** [**60**]	**Randomized, double-blind, placebo-controlled study**	***N*** **= 21**	**6 weeks**	**1. Group: CBD 75 mg/day**	**1. UPDRS total**	**Improvement of PDQ-39 with 300 mg CBD; UPDRS unchanged**	**None**
		**Age: 65.52 years**		**2. Group: CBD 300 mg/day**	**2. PDQ-39**		
					**3. UKU**		
		**Dd: 8.28 years**		**3. Group: placebo (corn oil)**	**4. Plasma BDNF levels**		
					**5. H1-MRS**		
**de Faria et al.** [**130**]	**Randomized, double-blind, placebo-controlled**	***N*** **= 24**	**Two experimental sessions within a 15-day interval**	**1. CBD group: 300 mg of pure CBD dissolved in corn oil**	**1.2 SPST sessions**	**Improvement of anxiety and tremor in CBD vs placebo group**	**NR**
					**2. UPDRS**		
		**Age: NR**			**3. VAMS**		
	**Crossover study**			**2. Placebo group: corn oil capsules**	**4. SSPS**		
		**Dd: NR**			**5. Blood pressure and heart rate**		
					**6. Accelerometer**		
**de Almeida et al.** [**86**]	**Phase II/III, randomized double-blind, placebo-controlled clinical trial**	***N*** **= 33**	**14 weeks**	**CBD 75 or 300 mg/day placebo (corn oil)** **CBD dissolved in corn oil**	**1. CGI**	**CBD showed no difference to placebo for primary outcomes** **Significant improvement in average sleep satisfaction with 300 mg was noted**	**Epigastric pain**
		**Age: 57 years** **Dd: 9 years**			**2. PSQI** **3. ESS** **4. RBDSQ** **5. PDSS** **6. BAI** **7. SDS** **8. PAS** **9. PDQ-39** **10. UPDRS**		**Headache** **Drowsiness** **Sadness** **Dizziness**
		

**de Almeida et al.** [**87**]	**Phase II/III, randomized double-blind, placebo-controlled clinical trial**	***N*** **= 18**	**14 weeks**	**75 or 300 mg/day CBD or placebo**	**Restless Legs Syndrome Rating Scale**	**No reduction in the severity of RLS in PD patients with RBD**	**NR**

**Table A2:** Uncontrolled studies

References	Study design	Sample size	Study duration	Type of the drug and route of administration	Measures	Main findings	Adverse events
		Age					
		Disease duration					
Frankel et al. [164]	Case series	*N* = 5	NR	1. Smoked cannabis (1 g with 2.9% THC)	Webster scale	No improvement of tremor	Drowsiness or mild euphoria
		Age: NR					
		Dd: NR					
				2. Diazepam 5 mg orally			Mild unsteadiness occurred after diazepam
				3. l-DOPA/Carbidopa 250 mg/25 mg orally (Sinemet 275)			
				4. Apomorphine 1.5 mg subcutaneously			
Venderová et al. [162]	Open-label pilot	*N* = 85 of 339	—	84 patients with ½ teaspoon cannabis orally; 1 patient by inhalation	Anonymous questionnaire sent by mail	31% reported improvement of rest tremor, 45% of bradykinesia and 14% of LID	Unspecified
		Age: 65.7 yearsDd: 8.5 yeasr					
				52.9% daily			
**Zuardi et al. [165]**	**Open-label pilot**	***N*** **= 6**	**4 weeks**	**1st week:150 mg/day**	**1.BPRS**	**Improvement of psychiatric plus and minus symptoms, mild improvement**	**None**
				**2nd week:250 mg/day**	**2. PPQ**		
		**Age: 58.8 ± 14.9 years**		**3rd week: 325 mg/day**	**3. UPDRS total**		
				**4th week: 400 mg/day**	**4. CGI-I**		
		**Dd: 10.6 ± 3.7 years**		**CBD dissolved in corn oil**	**5. MMSE**		
				**Placebo group: corn oil capsules**	**6. FAB**		
**Chagas et al.** [**60**]	**Case series**	**4 patients with RBD**	**6 weeks**	**75 or 300 mg/day CBD**	**REM Sleep behavior disorder questionnaire**	**Significant improvements in sleep symptoms; Well tolerated**	**NR**
Finseth et al. [166]	Patient survey	*N* = 9	—	Past or current use of Cannabis	Self-administered questionnaire via e-mail	Benefits in mood (56%), sleep (56%), motor symptoms (22%), and quality of life (22%)	None
		Age: 49–75 years					
		Dd: 2–11 years					
Lotan et al. [167]	Case series	*N* = 22	Smoked cannabis for at least 2 months	Smoked cannabis, 0.5 g (with 2.9% THC)	UPDRS VAS Pain intensity scale Short-Form McGill Pain Questionnaire Medical Cannabis Survey National Drug and Alcohol Research Center Questionnaire	Improvement of tremor and bradykinesia and some non-motor symptoms	Short-term: hypoglycemia, dizzinessLong-term: somnolence, drowsiness, palpitations, bad taste
		Age: 65 ± 10.2 yearsDd: 7.3 ± 4.8 years					
	
	

Shohet et al. [168]	Open-label, observational	*N* = 20	Cannabis use for at least 10 weeks (median 14 weeks)	Vaporizer or smoking Cannabis (1 g), anticholinergic agents, dopamine agonists, amantadine, and rasagiline	1. UPDRS	Improvement in mean motor and pain scores in 18PD patients	NR
					2. PRI		
					3. Short-form McGill Pain Questionnaire 4.VAS		
		Age: 62.4 ± 9 years					
		Dd: 6.8 ± 3.5 years					
		Control: 12 Age: 70 ± 7.2 years					
		Dd: 6 ± 2.6 years					
Yust-Katz et al. [169]	Open-label	*N* = 114	—	Medical cannabis (MC), anti- Parkinson medication, pain killers (paracetamol, NSAID), opiates (Tramadol, Oxycodone)	1. UPDRS	MC significantly alleviated pain	NR
		Age: 68 ± 9.9 years			2. McGill test	No significant difference in different pain medications (pain killers vs opiates vs MC)	
		Dd: 6 ± 6 years			3. Structured questionnaire for pain typ		
Balash et al. [170]	Retrospective observational telephone survey	*N* = 47	Patients treated for at least 3 months with MC	Most means of cannabis administration was Smoking flowers and leaves or oil ingestion daily dose 0.9 ± 0.5 g	Structured questionnaire	Improvement in pain, sleep, mood, and significant reduction of falls were reported by a significant percentage of patients.	Cough (34.9%), anxiety, confusion, and hallucinations
		Age: 64.2 ± 10.8 years					
		Dd: 10.8 ± 8.3 years					
Kindred et al. [171]	Open anonymous web-based survey	*N* = 453	—	Most reported routes of administration: smoking cannabis edible	Self-reported scales: GNDS, NHP, ABC, FSS, IPAQ	Improvement on mood, memory, fatigue, and obesity status, and reduction of prescribed medication	NR
		Age: 61.1 ± 9.5 years					
				Smoked + edibles			

Rows in bold emphasis indicate studies using CBD.
